# Supplementary material for: Insights into a Novel and Efficient Microbial Nest System for Treating Pig Farm Wastewater
Source: Microorganisms. 2025 Mar 19;13(3):685. doi: 10.3390/microorganisms13030685 (PMC11946184; doi:10.3390/microorganisms13030685)
Supplement: Supplementary file 1 [file microorganisms-13-00685-s001.zip › microorganisms-3502613-supplementary.pdf]

Supplementary material

Insights into a novel and efficient microbial nest system for treating pig farm wastewater

Table S1 Parameter setting of three-dimensional fluorescence spectrometry

Parameter	Setting
Emission wavelength range	250 nm-600 nm
Excitation wavelength range	200 nm-500 nm
Emission slit width	5 nm
Excitation slit width	5 nm
Excitation bandwidth	5 nm
Emission bandwidth	5 nm
Scan speed	1200 nm/min
Integration time	1 s
Data point interval	1 nm
Response time mode	Automatic
Scanning spectrometer calibration	Auto-calibration

Note: All measurements were performed at room temperature.

Table S2 Parameter setting of infrared spectrometry

Parameter	Setting
Wavelength range	4000-400 cm^{-1}
Resolution	4 cm^{-1}
Scan speed	2.0 cm/s
Number of scans	32
Apodization function	Happ-Genze
Zero-filling factor	2
Data collection mode	Single-beam
Background correction	Air

Note: All measurements were performed at room temperature.

Figure Captions

Fig. S1 Changes of TC, TN, NH_4^+-N and NO_3^--N contents during MNS fermentation

Fig. S2 FTIR spectroscopy characteristics of different MNS fermentation stages.

Fig. S3 Analysis of alpha diversity of bacterial communities during fermentation of microbial nests. (a) Ace index; (b) Chao1 index; (c) Smithwilson index (d) Shannon index; (e) Simpson index; (f) Coverage index.

Fig. S4 Analysis of alpha diversity of fungal communities during fermentation of microbial nests. (a) Ace index; (b) Chao1 index; (c) Smithwilson index (d) Shannon index; (e) Simpson index; (f) Coverage index.

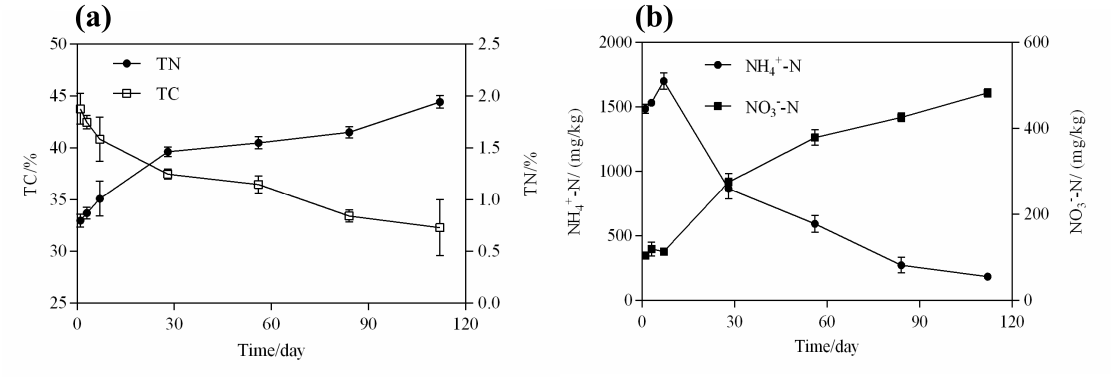

Fig. S1

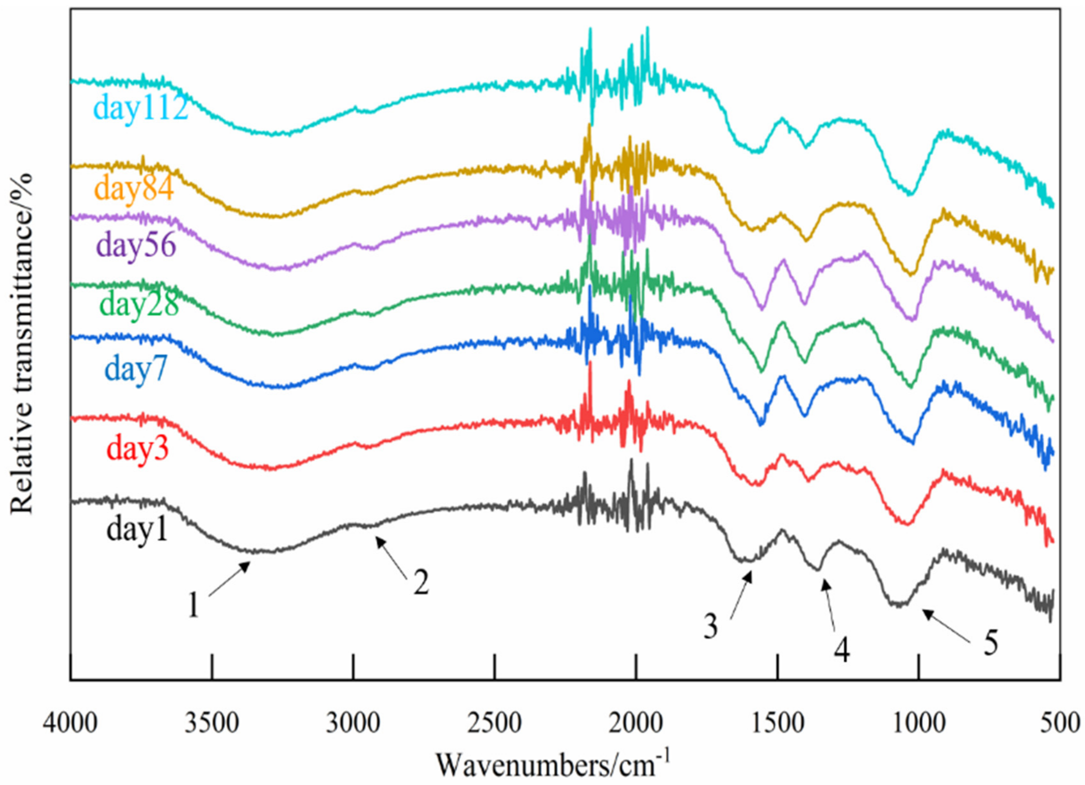
day112
day84
day56
day28
day7
day3
day1
1
2
3
4
5
Relative transmittance/%
4000
3500
3000
2500
2000
1500
1000
500
Wavenumbers/cm-1

Fig. S2

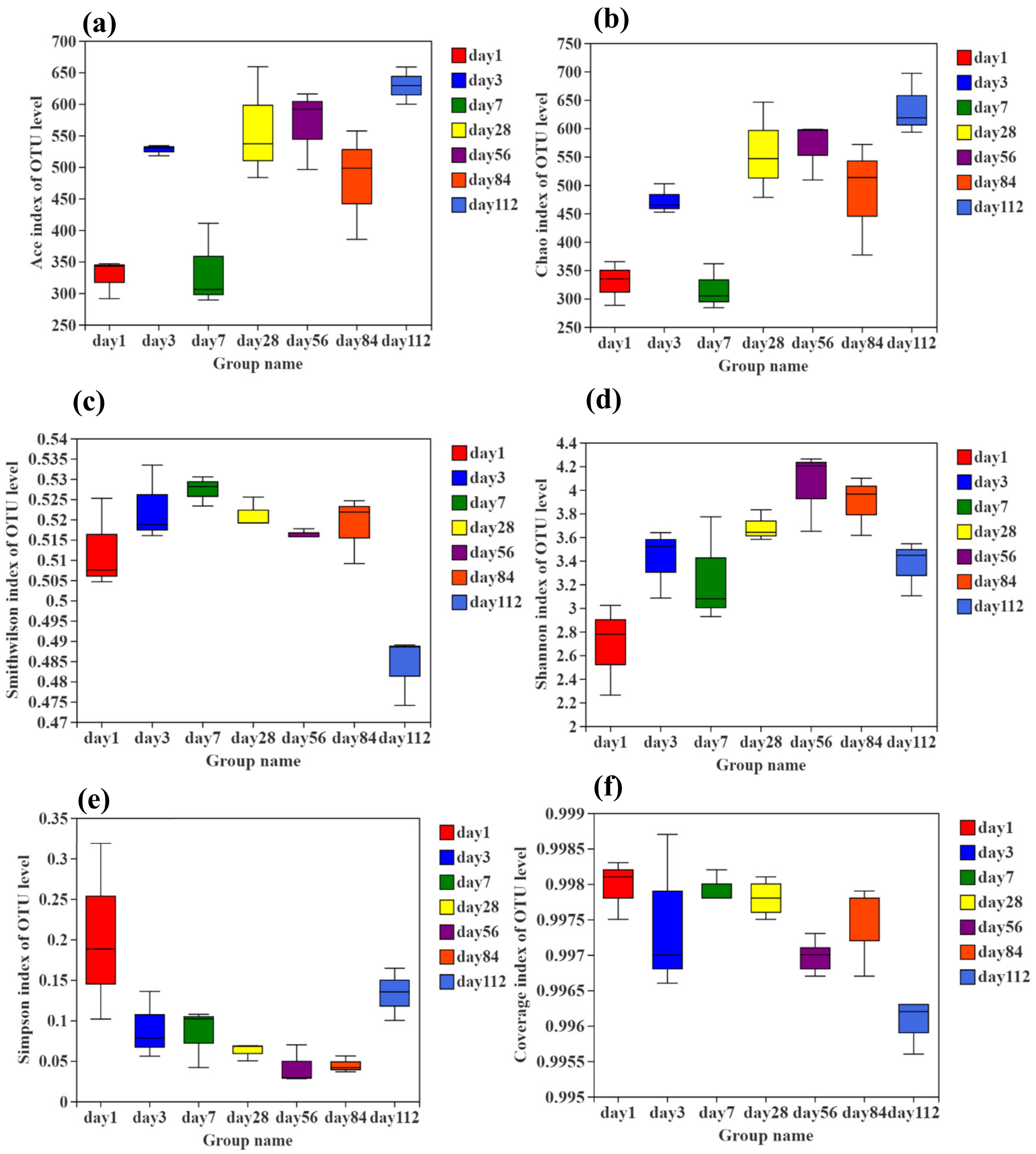
(a)
Ace index of OTU level
700
650
600
550
500
450
400
350
300
250
day1 day3 day7 day28 day56 day84 day112
Group name
day1
day3
day7
day28
day56
day84
day112
(b)
Chao index of OTU level
750
700
650
600
550
500
450
400
350
300
250
day1 day3 day7 day28 day56 day84 day112
Group name
day1
day3
day7
day28
day56
day84
day112
(c)
Smithwilson index of OTU level
0.54
0.535
0.53
0.525
0.52
0.515
0.51
0.505
0.5
0.495
0.49
0.485
0.48
0.475
0.47
day1 day3 day7 day28 day56 day84 day112
Group name
day1
day3
day7
day28
day56
day84
day112
(d)
Shannon index of OTU level
4.4
4.2
4
3.8
3.6
3.4
3.2
3
2.8
2.6
2.4
2.2
2
day1 day3 day7 day28 day56 day84 day112
Group name
day1
day3
day7
day28
day56
day84
day112
(e)
Simpson index of OTU level
0.35
0.3
0.25
0.2
0.15
0.1
0.05
0
day1 day3 day7 day28 day56 day84 day112
Group name
day1
day3
day7
day28
day56
day84
day112
(f)
Coverage index of OTU level
0.999
0.9985
0.998
0.9975
0.997
0.9965
0.996
0.9955
0.995
day1 day3 day7 day28 day56 day84day112
Group name
day1
day3
day7
day28
day56
day84
day112

Fig. S3

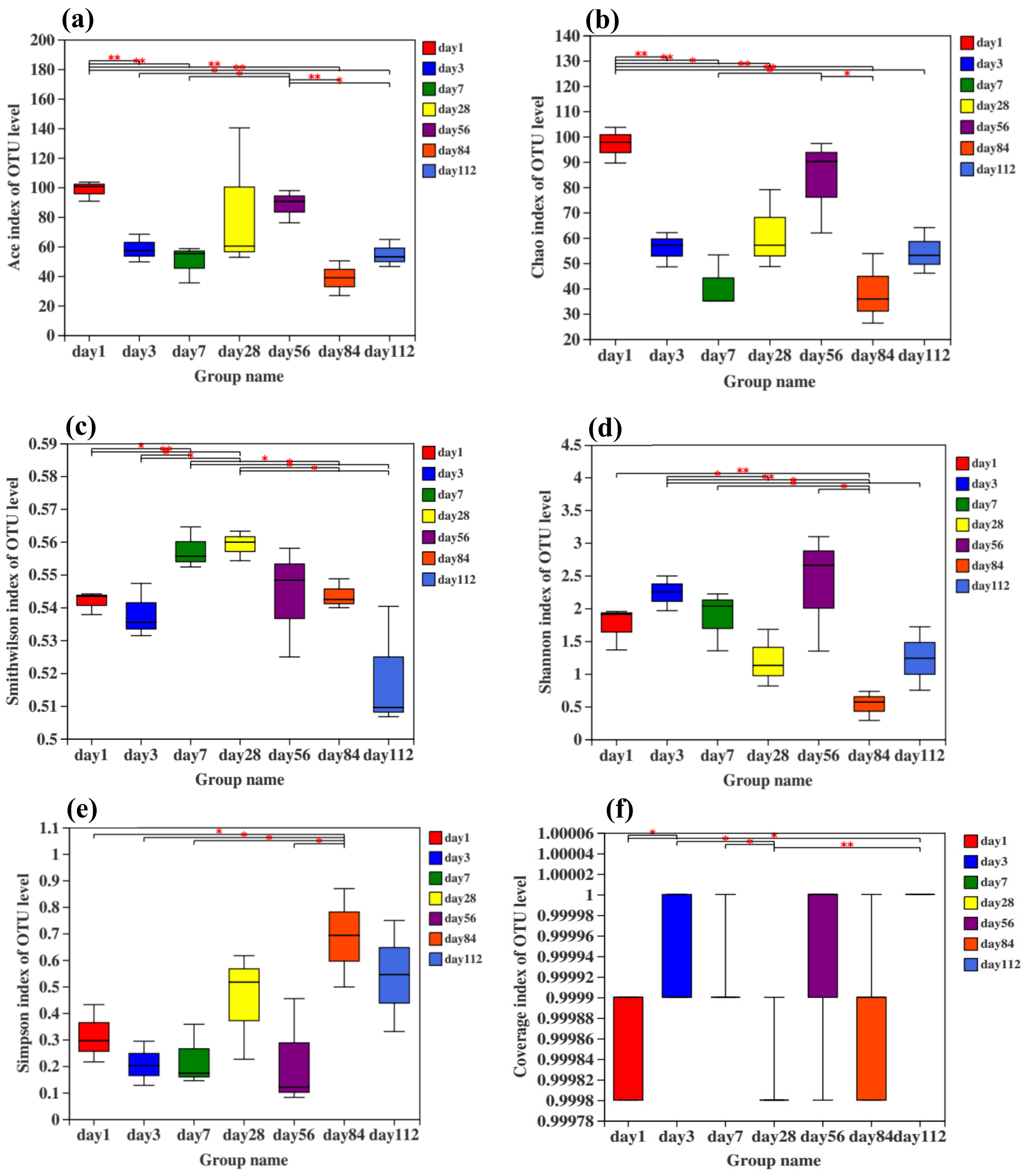
(a)
Ace index of OTU level
Group name
(b)
Chao index of OTU level
(c)
Smithwilson index of OTU level
(d)
Shannon index of OTU level
(e)
Simpson index of OTU level
(f)
Coverage index of OTU level
day1
day3
day7
day28
day56
day84
day112

Fig. S4
